# Honokiol activates LKB1-miR-34a axis and antagonizes the oncogenic actions of leptin in breast cancer

**DOI:** 10.18632/oncotarget.4937

**Published:** 2015-08-24

**Authors:** Dimiter B. Avtanski, Arumugam Nagalingam, Michael Y. Bonner, Jack L. Arbiser, Neeraj K. Saxena, Dipali Sharma

**Affiliations:** ^1^ Department of Oncology, Johns Hopkins University School of Medicine and the Sidney Kimmel Comprehensive Cancer Center at Johns Hopkins, Baltimore MD 21231; ^2^ Department of Medicine, University of Maryland School of Medicine, Baltimore MD 21201; ^3^ Department of Dermatology, Emory University School of Medicine, Winship Cancer Institute; ^4^ Atlanta Veterans Administration Medical Center, Atlanta, GA 30322

**Keywords:** Honokiol, leptin, LKB1, miR-34a, breast cancer

## Abstract

Leptin, a major adipocytokine produced by adipocytes, is emerging as a key molecule linking obesity with breast cancer therefore, it is important to find effective strategies to antagonize oncogenic effects of leptin to disrupt obesity-cancer axis. Here, we examine the potential of honokiol (HNK), a bioactive polyphenol from *Magnolia grandiflora*, as a leptin-antagonist and systematically elucidate the underlying mechanisms. HNK inhibits leptin-induced epithelial-mesenchymal-transition (EMT), and mammosphere-formation along with a reduction in the expression of stemness factors, Oct4 and Nanog. Investigating the downstream mediator(s), that direct leptin-antagonist actions of HNK; we discovered functional interactions between HNK, LKB1 and miR-34a. HNK increases the expression and cytoplasmic-localization of LKB1 while HNK-induced SIRT1/3 accentuates the cytoplasmic-localization of LKB1. We found that HNK increases miR-34a in LKB1-dependent manner as LKB1-silencing impedes HNK-induced miR-34a which can be rescued by LKB1-overexpression. Finally, an integral role of miR-34a is discovered as miR-34a mimic potentiates HNK-mediated inhibition of EMT, Zeb1 expression and nuclear-localization, mammosphere-formation, and expression of stemness factors. Leptin-antagonist actions of HNK are further enhanced by miR-34a mimic whereas miR-34a inhibitor results in inhibiting HNK's effect on leptin. These data provide evidence for the leptin-antagonist potential of HNK and reveal the involvement of LKB1 and miR-34a.

## INTRODUCTION

Given that one-third of all cancers are attributed to obese state, obesity is a well-established risk factor. Obese state is not only associated with aggressive tumor progression, poorer prognosis, increased recurrence and poorer survival of obese breast cancer patients, but it also impacts tumor initiation [1, 2]. Adipocytes lose their normal physiological size heterogeneity in obese state and undergo hypertrophy (increase in cell size) and hyperplasia (increase in cell number), leading to dysregulation of local and systemic secretion of biologically active polypeptides, adipocytokines such as leptin [3]. In recent years, leptin has emerged as a key candidate molecule mediating the molecular effects of obesity on cancer [4, 5]. Various epidemiological studies have shown that high level of plasma leptin is linked with increased risk and poor prognosis for breast carcinogenesis [6, 7]. Analysis of clinical samples showed overexpression of leptin receptor in 83% of breast tumor samples whereas no expression of leptin receptor was observed in normal mammary epithelial cells [8, 9]. Also, leptin overexpression was observed in 92% of breast tumors examined but in none of the cases of normal breast epithelium. Overexpression of leptin and leptin receptor in breast tumors and its association with tumor aggressiveness suggest that leptin can also influence breast tumor growth and progression via an autocrine pathway [9, 10].

Various research groups have been studying the oncogenic role of leptin in cancer. Studies from our lab and others have established that high leptin levels (hyperleptinemia) associated with obese state stimulate breast cancer cell proliferation, invasion, migration, and angiogenesis, thereby promoting breast tumor growth and metastasis [11–17]. Leptin also plays a central role in the acquisition of mesenchymal characteristics by inducing breast cancer cells to undergo a transition from epithelial to spindle-like mesenchymal morphology [13]. Leptin has been reported to regulate many signaling pathways and transcription factors implicated in breast cancer stem cells (BCSCs). Intact leptin-leptin receptor signaling was found to play an integral role in the survival of CSC population [18]. It is shown that leptin receptor is a characteristic feature of Tumor initiating stem cells (TISCs) and participates in the regulation of core pluripotency-associated transcription factors, Oct4 and Nanog [19, 20]. These studies indicate that leptin not only plays a significant role in promoting the growth and metastatic progression of established breast tumors but also augments tumor initiation and recurrence.

Leptin and leptin-signaling pathway have emerged as leading targets for disrupting obesity-breast cancer link; therefore, developing effective, non-endocrine, non-toxic agents for the inhibition of neoplastic effects of leptin is highly important. Current strategies to inhibit leptin pathway such as soluble Leptin receptors (LRs), synthetic leptin-antagonists, and anti-LR monoclonal antibodies (anti-LR mAbs) [17] are limited by associated toxicities as well as low efficacy. Recently, active constitutive agents in natural products used in traditional Asian medicine have shown efficacy as potential cancer preventive as well as therapeutic agents [21, 22]. For years, cones, bark and leaves from *Magnolia* plant species have been used for their anti-thrombocytic, anti-inflammatory, anxiolytic, anti-depressant, antioxidant, antispasmodic, and antibacterial effects [23–26]. It is now known that Honokiol (HNK), a natural phenolic compound isolated from an extract of seed cones from *Magnolia grandiflora* [27] is responsible for these medicinal benefits of Magnolia species. Previous studies from our lab have shown that HNK inhibits breast carcinogenesis *in vitro* and *in vivo* [28, 29] thereby establishing HNK as a promising bioactive compound against breast carcinogenesis. We also found that honokiol treatment increases the expression of tumor suppressor LKB1 which plays an integral role in honokiol-mediated inhibition of breast tumor growth and progression [28]. Recently, we discovered the involvement of miR-34a in breast tumor inhibition function of honokiol. Honokiol treatment inhibited breast tumor growth in lean and obese-hyperleptinemic mice models in a manner associated with activation of miR-34a [30]. In this report, we specifically investigated the potential of HNK to inhibit leptin-induced epithelial-mesenchymal transition (EMT) and tumorsphere formation and examine the underlying molecular mechanisms. We provide molecular evidence supporting the regulatory role of LKB1, and integral involvement of miR-34a in leptin-antagonist potential of HNK.

## RESULTS

### Honokiol inhibits leptin-induced epithelial-mesenchymal transition, mammosphere formation, and migration of breast cancer cells

**Figure 1:**
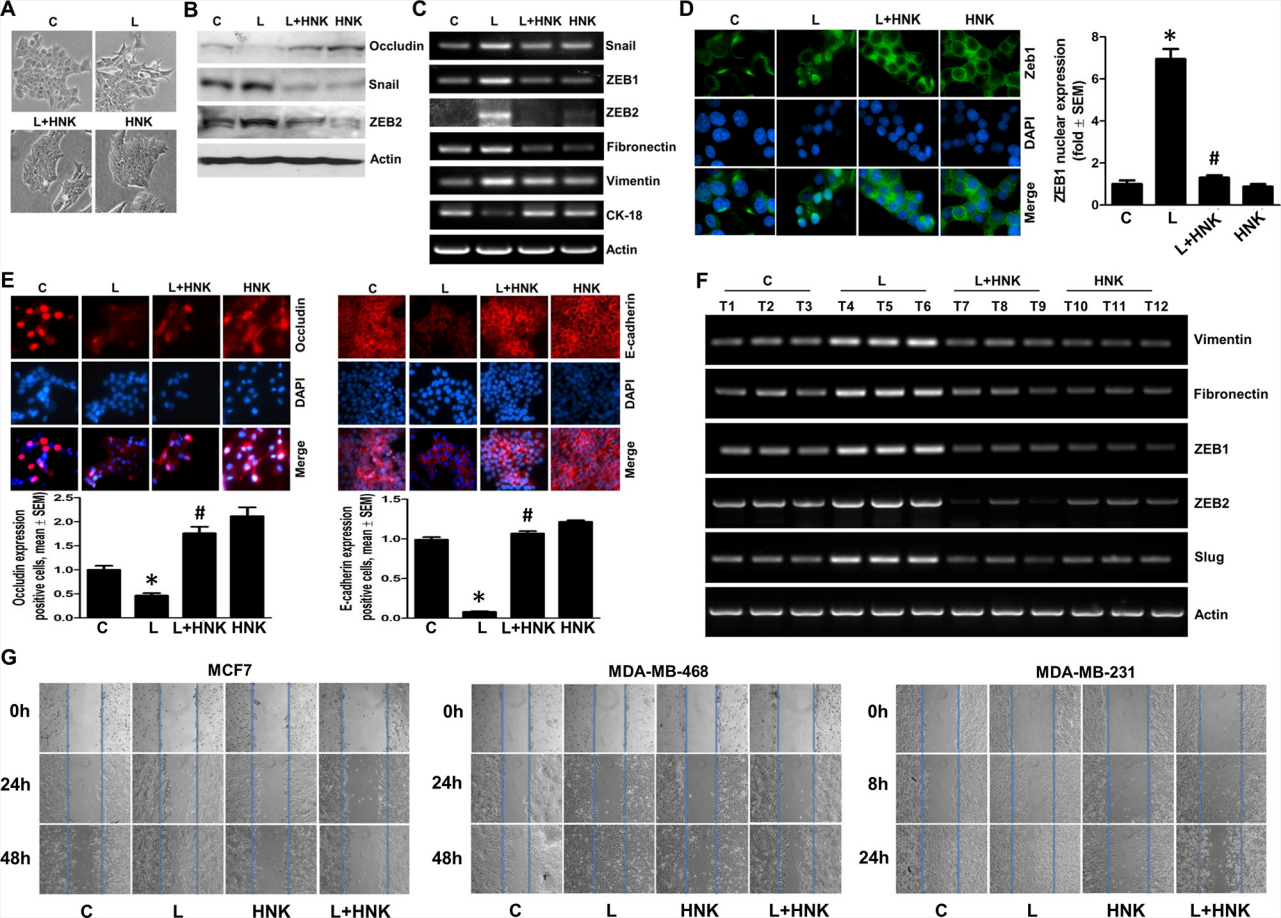
Honokiol inhibits leptin-induced epithelial-mesenchymal transition and migration of breast cancer cells **A.** MCF7 cells were treated with leptin (L) (100 ng/ml) and/or Honokiol (HNK) (5 μM) as indicated. Vehicle treated cells are denoted as (C) Control. Morphological changes associated with EMT are shown in phase-contrast images. The presence of spindle-shaped cells, increased intracellular separation and pseudopodia were noted in leptin-treated cells but not in HNK-treated cells. **B.** MCF7 cells were treated as in A and total lysates were immunoblotted for Occludin, Snail and Zeb2 expression levels. Actin was used as control. **C.** MCF7 cells were treated as in A, total RNA was isolated and expression levels of epithelial and mesenchymal marker genes was analyzed. Actin was used as control. **D.** Breast cancer cells were treated as in A, and subjected to immunofluorescence analysis (1000X magnification) of Zeb1. Bar-graphs show the fold-change in number of cells expressing nuclear Zeb. *p < 0.001, compared with untreated controls. #*p* < 0.001, compared with leptin-alone treatment. Leptin-induced nuclear translocation of Zeb1 was abrogated by HNK treatment. **E.** Breast cancer cells were treated as in A, and subjected to immunofluorescence analysis (200X magnification) of E-cadherin, and Occludin. Bar-graphs show the fold-change in number of cells expressing Occludin and E-cadherin. *p < 0.05, compared with untreated controls. #*p* < 0.01, compared with leptin-alone treatment. **F.** MDA-MB-231 cells derived tumors were developed in nude mice and treated with leptin and/or HNK (*n* = 6–8/treatment group). At the end of five weeks of treatment, tumors were collected. Total RNA was isolated from tumor samples and subjected to RT-PCR analysis for the expression of mesenchymal markers and transcription factors. **G.** Breast cancer cells were treated as in A and subjected to scratch-migration assay.

Epithelial to mesenchymal transition (EMT) of cancer cells is a crucial early event leading to induction of cell motility, invasion and distant metastasis. We recently presented a pivotal role of leptin in acquisition of mesenchymal characteristics and aggressive behavior in breast cancer cells [13]. Here, we specifically examined if HNK could inhibit the stimulatory effect of leptin on EMT and metastatic properties of breast cancer cells. Following treatment with leptin and HNK, we observed striking morphological differences between MCF7 cells treated with different combinations. Leptin-treated MCF7 cells exhibited acquisition of fibroblast-like appearance and increased formation of pseudopodia observed emanating from the cell membrane. These features signify typical mesenchymal phenotype rather than the normal epithelial phenotype of MCF7 cells, showing that cells have undergone EMT upon leptin treatment. HNK prevented the morphological transition from an epithelial-like to mesenchymal-like appearance caused by leptin treatment. HNK alone did not affect the morphology of MCF7 cells (Figure 1A). To unequivocally establish that HNK blocks leptin-induced EMT, we next examined the biochemical hallmarks of EMT-reversal including gain of expression of epithelial markers (occludin, and cytokeratin-18 (CK-18) with a concomitant decrease in mesenchymal markers (fibronectin, and vimentin) expression. Leptin treatment resulted in upregulation of mesenchymal markers accompanied with a marked decrease in the expression of epithelial markers. HNK blocked leptin-induced modulation of mesenchymal and epithelial markers leading to decreased expression of fibronectin and vimentin and increased expression of CK-18 and occludin (Figure 1B and 1C, Supplementary Figure 1A). Immunocytochemical analysis provided additional evidence to support HNK-mediated leptin-induced EMT reversal showing gain of expression of occluding and E-cadherin (Figure 1E). Transcriptional repressors for epithelial marker proteins, Zeb1/2 and snail, are frequently detected in metastatic cancer cells and are known to be involved in EMT [31, 32]. We also examined the involvement of these transcription repressors in HNK-mediated inhibition of leptin-induced EMT. Indeed, leptin treatment not only increased the expression of snail, Zeb1 and Zeb2 but also increased the nuclear translocation of Zeb1 (Figure 1B, 1C and 1D). Importantly, HNK treatment inhibited leptin-induced expression of snail, Zeb1 and Zeb2 as well as promoted cytoplasmic retention of Zeb1 even in the presence of leptin (Figure 1B, and 1D). In a recent study, we showed that HNK administration retarded leptin-induced growth of MDA-MB-231 cells implanted in female athymic mice [30]. We used tumor samples from the same study to evaluate the effect of HNK on leptin-induced mesenchymal markers by RT-PCR analysis. Corroborating *in vitro* findings, tumors from mice co-treated with HNK and leptin showed decreased levels of expression of vimentin, fibronectin, Zeb1/2 and slug in comparison to tumors from leptin-treated mice (Figure 1F). Since HNK inhibited leptin-induced EMT, we aimed to examine whether HNK treatment also blocked induction of migration usually observed in the presence of leptin. Significant migration of MCF7, MDA-MB-468, MDA-MB-231, SUM149, SUM159 and T47D breast cancer cells observed in the presence of leptin was inhibited in the presence of HNK treatment (Figure 1G, Supplementary Figure 1B, 1C). Honokiol treatment does not affect growth of MCF10A cells while leptin show only modest effects on MCF10A cells (Supplementary Figure 2).

**Figure 2:**
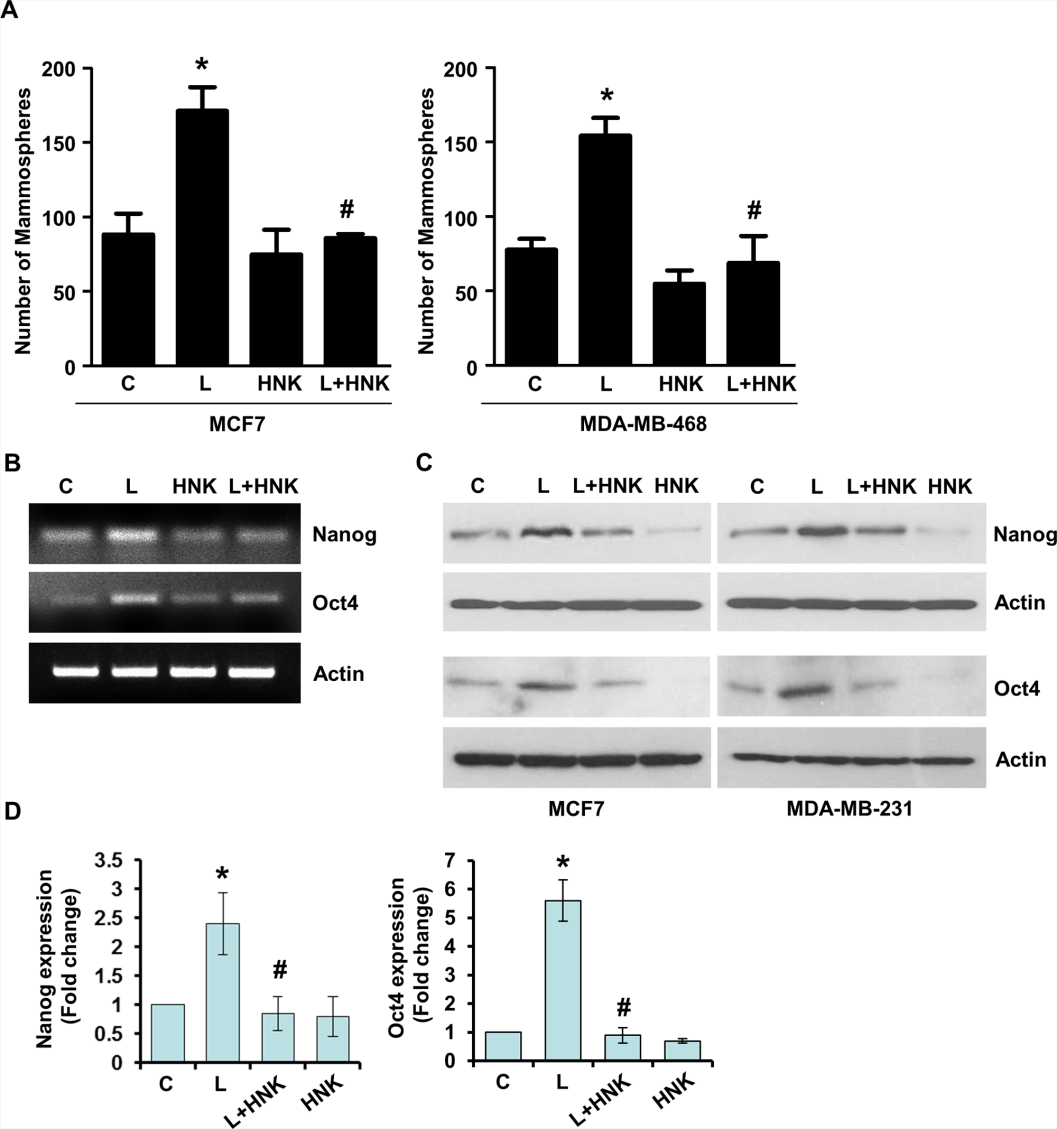
Honokiol abates the stimulatory effect of leptin on mammosphere-formation-potential and acquisition of stem-like properties in breast cancer cells **A.** MCF7 and MDA-MB-468 cells were treated with leptin (L) (100 ng/ml) and/or Honokiol (HNK) (5 μM) as indicated and subjected to mammosphere formation. Vehicle-treated cells are denoted as (C) The graph shows the number of mammospheres. *p < 0.001, leptin treatment compared with untreated controls. # *p* < 0.01, leptin+honokiol compared with leptin-alone treatment. **B.** MCF7 cells were treated with leptin (100 ng/ml) and/or HNK (5 μM) alone or in combination as indicated, total RNA was isolated and expression levels of Nanog and Oct4 were examined. Actin was used as control. **C.** Breast cancer cells were treated as in B, and total lysates were immunoblotted for Nanog and Oct4 expression levels. Actin was used as control. **D.** Bar-graphs show the fold-change in Nanog and Oct4 expression in breast cancer cells treated with leptin and/or honokiol. *p < 0.001, leptin treatment compared with untreated controls. #*p* < 0.001, leptin+honokiol compared with leptin-alone treatment.

An emerging hypothesis is that EMT bestows cells with stem-like characteristics and facilitates an increase in the subpopulation of CSC (cancer stem cells) [33–35]. Utilizing mammosphere assay that relies on the unique property of breast cancer cells with stem-like potential to form large, round, unattached floating spheroid colonies (termed mammosphere), we showed that leptin induced mammosphere formation. HNK treatment efficiently inhibits leptin-induced EMT as well as migration of breast cancer cells, therefore, we hypothesize that HNK may also inhibit leptin-induced mammosphere formation. Indeed, HNK inhibited mammosphere formation even in the presence of leptin (Figure 2A). Given the association of induced pluripotent stem cell (iPSC) markers, Nanog and Oct4 with self-renewal and maintenance of stem cell fate, we wished to investigate whether iPSC markers are affected by leptin treatment. To this end, we assessed the expression of Oct4, and Nanog in leptin-treated breast cancer cells and found that the expression of pluripotency genes was higher in leptin-treated cells (Figure 2B–2D) with respect to the expression in untreated cells. Cells treated with HNK exhibited reduced levels of iPSC markers and interestingly, HNK treatment resulted in the inhibition of leptin-induced expression of Oct4 and Nanog (Figure 2B–2D). Breast cancer cells treated with leptin and honokiol were examined for the expression of Cyclin D1 as a functional control (Supplemental Figure 3). Together, these results show that HNK treatment results in effective inhibition of leptin-induced epithelial-mesenchymal transition, mammosphere formation, stemness and migration of breast cancer cells.

### Honokiol induces expression and cytoplasmic localization of tumor suppressor Liver Kinase B1 in breast cancer cells

**Figure 3:**
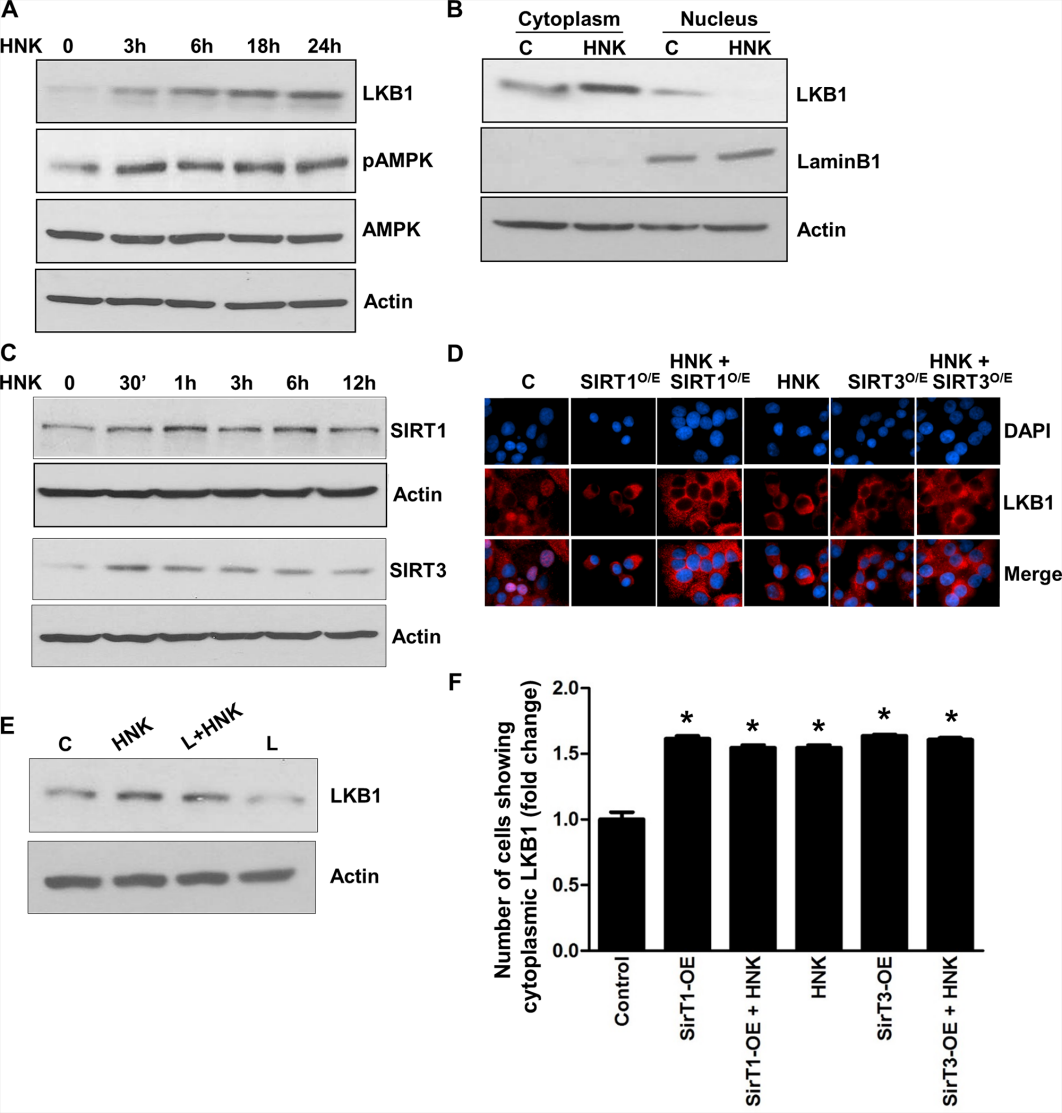
Honokiol induces the expression and cytoplasmic localization of tumor suppressor LKB1 and involvement of SIRT1/3 **A.** MCF7 cells were treated with 5 μM HNK for various time intervals as indicated, total lysates were immunoblotted for LKB1, phospho-AMPK and total AMPK expression levels. Actin was used as control. **B.** MCF7 cells were treated with 5 μM HNK for 6 h, nuclear and cytoplasmic fractions were immunoblotted for LKB1 expression. Actin was used as control. **C.** MCF7 cells were treated with 5 μM HNK for various time intervals as indicated, total lysates were immunoblotted for SIRT1 and SIRT3 expression levels. Actin was used as control. **D.** MCF7 cells were transfected with SIRT1 and SIRT3 overexpression constructs as indicated followed by 5 μM HNK treatment. Cells were subjected to immunofluorescence analysis of LKB1. **E.** MCF7 cells were treated with leptin (100 ng/ml) and/or HNK (5 μM) as indicated, cell lysates were immunoblotted for LKB1. **F.** Bar-graphs show the fold-change in number of cells expressing cytoplasmic localization of LKB1.

Liver kinase B1 (LKB1), owing to its dual role as a tumor suppressor and an upstream master kinase, serves as a major hub regulating several downstream pathways and known tumor suppressors (e.g., AMPK-mTOR, JNK and p53) [36, 37]. We found that HNK increases expression of LKB1 in breast cancer cells (Figure 3A). Interestingly, leptin treatment inhibits LKB1 expression whereas HNK treatment increases LKB1 expression even in the presence of leptin (Figure 3E). Since LKB1 has recently been identified as a critical upstream kinase for AMPK regulating its activity, we examined the phosphorylation and expression of AMPK. Honokiol-treatment increased phosphorylation and expression of AMPK (Figure 3A). Tumor-suppressor function of LKB1 is majorly attributed to the cytoplasmic pool of LKB1 as mutant LKB1 lacking the nuclear localization signal still retains the ability to suppress cell growth [38, 39]. Examining nuclear and cytoplasmic fractions of breast cancer cells treated with HNK, we found that HNK induced cytoplasmic localization of LKB1 (Figure 3B). Recent studies have reported an important role of Sirtuin-deacetylases, SIRT1 and SIRT3 in LKB1 regulation. SIRT1 and SIRT3 overexpression diminishes lysine acetylation of LKB1 and concurrently increases its activity and cytoplasmic/nuclear ratio [40, 41]. We found that breast cancer cells treated with HNK exhibited an increase in the expression of SIRT1 and SIRT3 within 30 minutes post-treatment (Figure 3C). To examine the effect of SIRT1 and SIRT3 on HNK-mediated cytoplasmic localization of LKB1, we overexpressed SIRT1 and SIRT3 in MCF7 cells followed by HNK treatment. Immunofluorescence analysis showed that LKB1 is present both in nucleus and cytoplasm in untreated cells. Corroborating immunoblot analyses shown in figure 3B, HNK-treated cells exhibited increased cytoplasmic localization of LKB1 in comparison to untreated cells which was further enhanced with SIRT1 and SIRT3 overexpression (Figure 3D, 3F)). Our studies show a role of sirtuin-deacetylases in HNK-mediated functional upregulation of LKB1.

### Honokiol upregulates miR-34a in a LKB1-dependent manner

**Figure 4:**
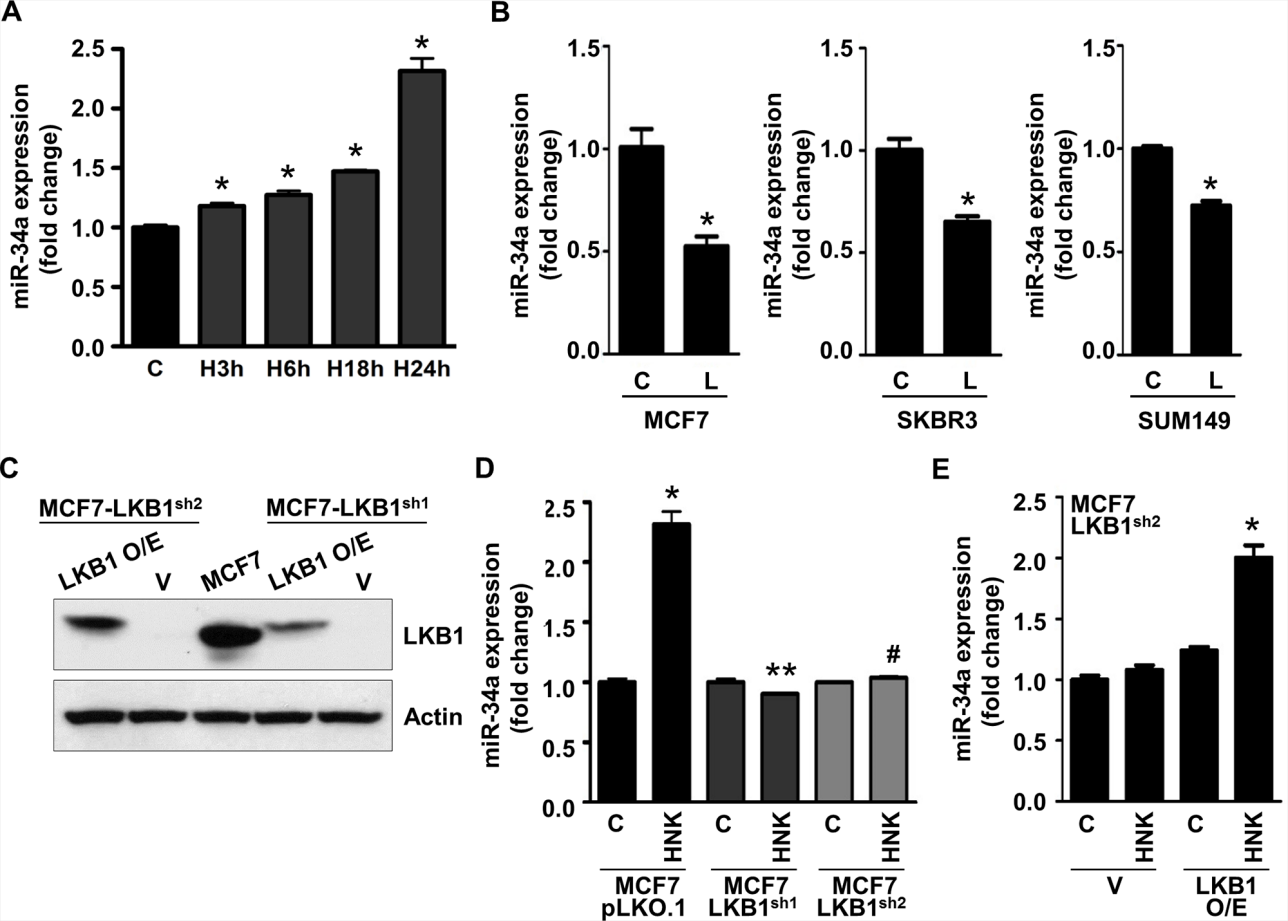
Honokiol upregulates miR-34a in LKB1-dependent manner in breast cancer cells **A.** Expression levels of miR-34a in MCF7 cells treated with 5 μM HNK for various time intervals as indicated. *p < 0.001, compared with untreated controls. **B.** MCF7, SKBR3 and SUM149 cells were treated with 100 ng/ml leptin and miR-34a expression levels were measured. *p < 0.005, compared with untreated controls. **C.** Immunoblot analysis of LKB1 in stable pools of LKB1-depleted (LKB1^shRNA 1–2^) and vector control (pLKO.1) MCF7 cells. LKB1-depleted (LKB1^shRNA 1–2^) were transfected with LKB1 overexpression vector to create a ‘gain-of-function’ system. **D.** Expression levels of miR-34a in stable pools of LKB1-depleted (LKB1^shRNA 1–2^) and vector control (pLKO.1) MCF7 cells. *p < 0.001, compared with untreated controls; **p < 0.001, compared with HNK-treated MCF7-pLKO.1 cells; #*p* < 0.001, compared with HNK-treated MCF7-pLKO.1 cells. **E.** LKB1-depleted (LKB1^shRNA2^) were transfected with LKB1 overexpression vector and expression levels of miR-34a was examined. *p < 0.001, compared with HNK-treated, control-transfected, MCF7- LKB1^shRNA2^ cells.

Regulatory role of microRNAs (miRNA) in various biological and pathological processes, including cancer progression and metastasis has been well-recognized in recent years. Functionally, miRs are small (∼21-mer) regulatory RNA molecules that exert their regulatory effects by binding to the 3′-untranslated regions (3′-UTR) of specific mRNAs triggering mRNA degradation or translational repression [42]. miRNAs have been known to function both as oncogenes, potentiating cancer progression and metastasis, and tumor suppressors implicated in growth inhibition [43]. One of the important tumor suppressor miRNA involved in breast cancer is miR-34a, which is downregulated in aggressive breast tumors [44]. Breast cancer cells treated with HNK exhibited a time-dependent increase in miR-34a expression (Figure 4A). Interestingly, leptin treatment decreased the expression of miR-34a in MCF7, SKBR3 and SUM149 breast cancer cells (Figure 4B). We raised the question whether LKB1 plays any role in honokiol-mediated increase of miR-34a. We used LKB1^shRNA^ lentivirus and puromycin to select for stable pools of MCF7 cells with LKB1 depletion. pLKO.1 and LKB1^shRNA^ stable MCF7 cell pools were analyzed for LKB1 protein expression by immunoblot analysis. LKB1 protein expression was significantly reduced in LKB1^shRNA^ cells (shRNA1 and shRNA2) as compared to pLKO.1 control cells (Figure 4C). pLKO.1 and LKB1^shRNA^ cells were treated with HNK and expression of miR-34a was determined. Intriguingly, displaying a crucial role of LKB1, honokiol treatment did not increase miR-34a expression in LKB1^shRNA^ cells while pLKO.1 cells exhibited HNK-induced miR-34a expression (Figure 4D). As a gain-of-function strategy, LKB1 was overexpressed in LKB1^shRNA^ cells (Figure 4C), treated with HNK followed by examination of miR-34a expression. As evident in Figure 4E, HNK treatment increased miR-34a expression in LKB1^shRNA^ cells overexpressing LKB1. These results show that LKB1 plays an important role in honokiol-induced miR-34a expression in breast cancer cells.

### Honokiol inhibits EMT, stemness and leptin function in a miR-34a-dependent manner

**Figure 5:**
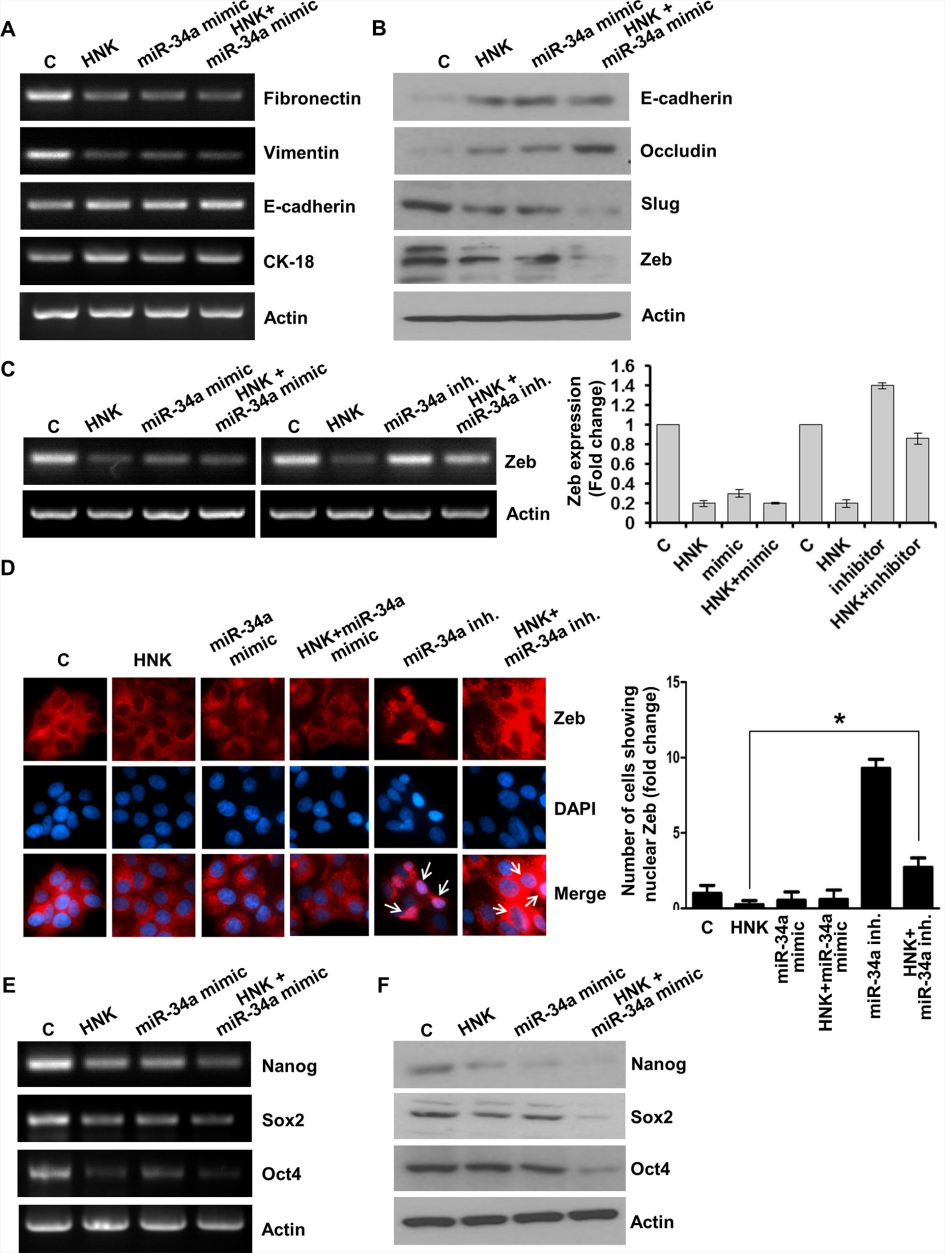
Evidence supporting the involvement of miR-34a in honokiol-mediated modulation of EMT and stemness factors **A.** MCF7 cells were transfected with miR-34a mimic followed by treatment with vehicle (C) or HNK (5 μM) as indicated, total RNA was isolated and expression levels of epithelial and mesenchymal marker genes was analyzed. Actin was used as control. **B.** MCF7 cells were transfected with miR-34a mimic followed by treatment with vehicle **C.** or HNK (5 μM) as indicated, total protein lysates were immunoblotted for the expression levels of epithelial and mesenchymal marker genes as indicated. Actin was used as control. (C) MCF7 cells were transfected with miR-34a inhibitor or miR-34a mimic followed by treatment with vehicle (C) or HNK (5 μM) as indicated, total RNA was isolated and expression levels of Zeb1 was analyzed. Actin was used as control. Bar-graph shows fold-change in Zeb expression. **D.** MCF7 cells were transfected with miR-34a inhibitor or miR-34a mimic followed by treatment with vehicle (C) or HNK (5 μM) as indicated, immunofluorescence analysis for Zeb1 was performed. Arrows point the cells showing nuclear localization of Zeb1. Bar-graphs show the fold-change in number of cells expressing nuclear localization of Zeb1. *p < 0.02, HNK treated cells compared with HNK+miR-34a inhibitor treated cells. **E.** MCF7 cells were transfected with miR-34a mimic followed by treatment with vehicle (C) or HNK (5 μM) as indicated, total RNA was isolated and expression levels of stemness genes (Oct4, Nanog, Sox2) was analyzed. Actin was used as control. **F.** Breast cancer cells were transfected with miR-34a mimic followed by treatment with vehicle (C) or HNK (5 μM) as indicated, total protein lysates were immunoblotted for the expression levels of stemness genes (Oct4, Nanog, Sox2) genes as indicated. Actin was used as control.

We further investigated the importance of miR-34a in HNK-mediated modulation of EMT markers, stemness factors and inhibition of leptin function. Ectopic miR-34a expression (in the form of a mimic molecule) further enhanced HNK-mediated inhibition of mesenchymal markers Fibronectin and Vimentin while enhancing the expression of epithelial markers, E-cadherin, Occludin and CK-18 (Figure 5A, 5B). HNK treatment decreased expression of slug and Zeb1, repressors of E-cadherin in comparison to untreated cells. Combination of HNK and miR-34a mimic further enhanced the inhibition slug and Zeb1 (Figure 5B, 5C). Expression of miR-34a-inhibitor increased Zeb1 expression even in the presence of HNK (Figure 5C). Next, we investigated the effect of miR-34a mimic and inhibitor on HNK-induced cytoplasmic retention of Zeb1 in breast cancer cells. Breast cancer cells treated with HNK, miR-34a mimic alone and in combination exhibited cytoplasmic localization of Zeb1. Treatment with miR34a inhibitor caused nuclear localization of Zeb1 (Figure 5D). To investigate the involvement of miR-34a in HNK-mediated inhibition of iPSC factors, we treated breast cancer cells with HNK in the presence of miR34a mimic or inhibitior. As expected, HNK inhibited the expression of Oct4, Nanog and Sox2 in breast cancer cells which is further decreased by ectopic miR-34a expression (Figure 5E, 5F).

**Figure 6:**
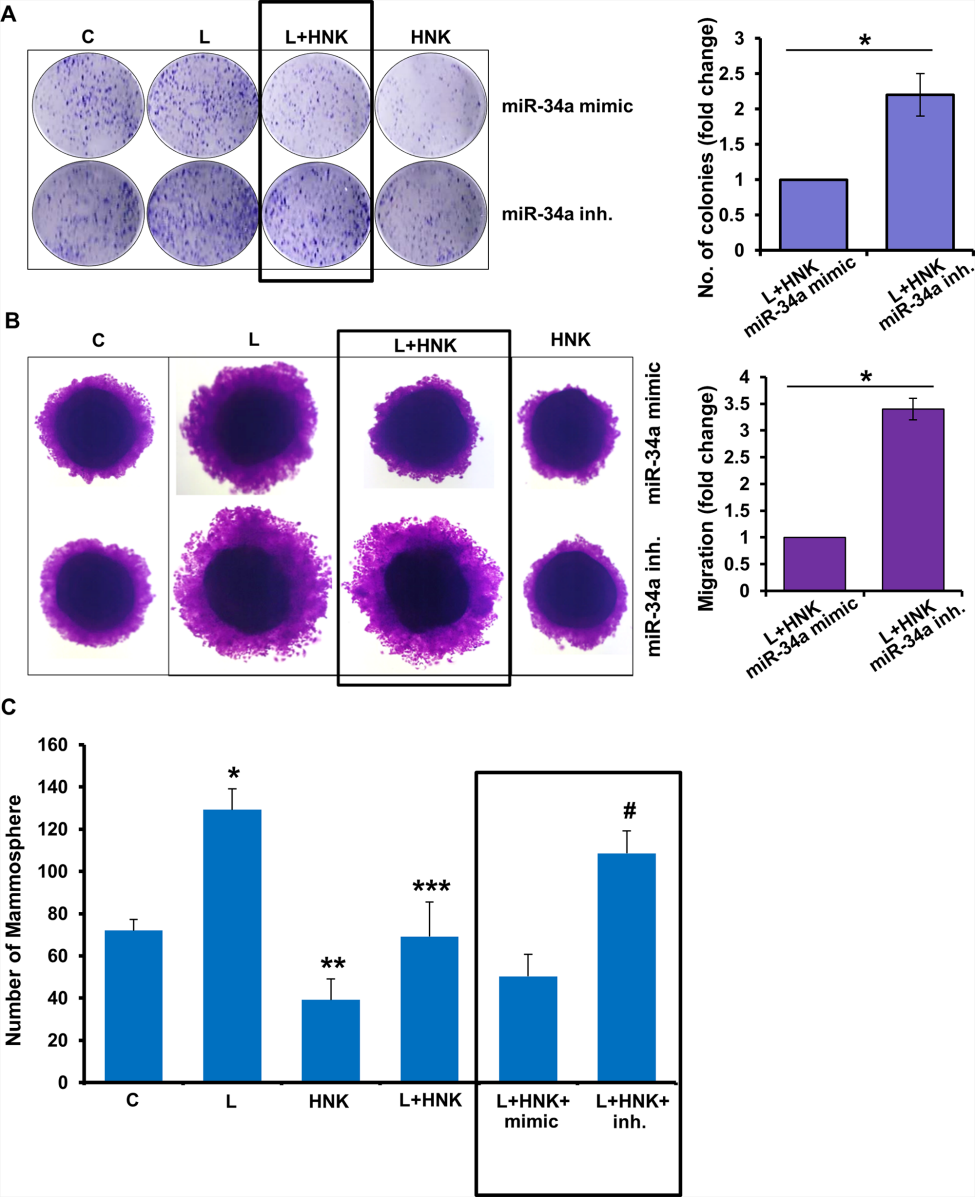
Role of miR-34a in honokiol-mediated inhibition of oncogenic actions of leptin **A.** MCF7 cells were transfected with miR-34a inhibitor or miR-34a mimic followed by treatment with vehicle (C), Honokiol (HNK) (5 μM) and/or leptin (L) (100 ng/ml) as indicated and subjected to (A) clonogenicity assay, **B.** spheroid migration assay and **C.** mammosphere assay. *p < 0.05, L+HNK+miR-34a mimic treated cells compared with L+HNK+miR-34a inhibitor treated cells.

Given our results showing that miR-34a plays an important role in HNK-mediated modulation of EMT markers and iPSC factors, we decided to examine whether miR-34a modulation also affects HNK-mediated inhibition of oncogenic actions of leptin. Expression of miR-34a mimic potentiated HNK-mediated-inhibition of leptin-induced clonogenicity, migration and mammosphere formation. On the other hand, miR-34a inhibitor interfered with HNK efficacy as a leptin-antagonist resulting in poor inhibition of leptin-induced clonogenicity, migration and mammosphere formation (Figure 6A, 6B, 6C). Leptin treatment decreases miR-34a expression (Figure 4B), therefore, it is interesting to note that miR-34a mimic reduced oncogenic effects of leptin while miR-34a inhibitor increased leptin's impact (Figure 6A, 6B, 6C). Together, these data provide evidence supporting an important role of miR-34a in HNK-mediated inhibition of oncogenic actions of leptin.

**Figure 7:**
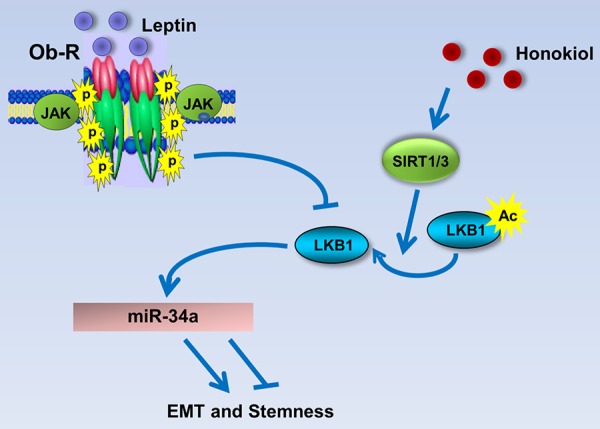
Schematic representation of the mechanism whereby HNK inhibits leptin-induced EMT and stemness via LKB1 and miR-34a Leptin treatment inhibits LKB1 expression. Honokiol treatment induces the expression levels of SIRT1/3 and stimulates the expression as well as cytoplasmic localization of LKB1 and also increases the levels of miR-34a leading to the inhibition of EMT and stemness markers. Honokiol treatment results in the inhibition of EMT, migration and mammosphere formation even in the presence of leptin.

Collectively, the findings presented here suggest that HNK inhibits leptin-induced EMT, stemness and mammosphere formation, and provide evidence for the involvement of miR-34a as a regulator of HNK-mediated inhibition of oncogenic actions of leptin, and uncover a novel mechanism of HNK action through activation of miR-34a in a LKB1-dependent manner (Figure 7).

## DISCUSSION

Over the last decade, there has been growing emphasis on the importance of EMT, an essential normal physiological process for embryonic development, tissue remodeling and wound healing, now implicated in cancer progression. An oncogenic EMT causes epithelial-derived tumors to gain a mesenchymal phenotype facilitating migration and invasion potential of cancer cells. Acquisition of mesenchymal traits not only promotes dissemination of cancer cells from primary tumors, increasing metastatic progression but is also linked with other pro-metastatic traits such as increase in tumor-initiating cell (TIC)-characteristics including self-renewal, multipotency and resistance to conventional therapeutics [33–35]. Recent studies from our lab and others have shown an important role of leptin in acquisition of mesenchymal characteristics, and survival of cancer stem cells (CSCs) *in vitro* and *in vivo* [13, 20]. Striving to develop effective, clinically viable leptin-antagonists using bioactive compounds, we recently discovered that HNK is capable of inhibiting breast cancer growth in hyperleptinemic state [30]. These discoveries sparked our interest in investigating the efficacy of HNK as a novel inhibitor of leptin-induced EMT and stemness in breast cancer. We found that HNK effectively inhibits leptin-induced EMT of breast cancer cells as evident from morphological changes and molecular alterations of mesenchymal and epithelial genes. Breast tumors treated with HNK also showed reduced expression of mesenchymal markers even in the presence of leptin treatment providing *in vivo* evidence. Mammosphere formation induced by leptin was also efficiently abrogated with HNK treatment along with inhibition of expression of iPSC factors (Oct4, nanog and Sox2). These findings imparted convincing evidence supporting the efficacy of HNK as a novel inhibitor of leptin-induced EMT and stemness warranting further mechanistic investigations. Consequently, we designed further studies to decipher the key nodes of leptin-antagonist function of HNK to facilitate establishing surrogate biomarkers for its efficacy and help in clinical development of this bioactive molecule as a leptin-antagonist. We found that HNK induces expression and cytoplasmic localization of LKB1. HNK also increases expression of Sirtuin-deacetylases, SIRT1/3 and overexpression of both SIRT1 and SIRT3 further increases cytoplasmic localization of LKB1. HNK upregulates miR-34a in a LKB1-dependent manner; miR-34 a plays an important role in HNK-mediated inhibition of EMT, stemness and leptin-function.

Tumor suppressor LKB1, a key determinant in Peutz-Jeghers syndrome, has been found to be inactivated in a subset of sporadic lung and pancreatic cancer [37, 45]. LKB1 inactivation is not commonly correlated with human breast carcinoma but interestingly, LKB1 loss has been observed in high-grade DCIS and high-grade invasive ductal carcinoma [46]. Importantly, LKB1 expression was abrogated only in the DCIS associated with invasion but not in pure DCIS cases indicating that LKB1 loss might promote invasive behavior. In fact, low LKB1 protein levels correlate with poor prognosis in breast carcinoma [47]. LKB1 knockdown increases motility and invasiveness of cancer cells, and induces the expression of many mesenchymal marker proteins indicating its possible role in EMT [48, 49]. We show that HNK increases the expression of LKB1 in breast cancer cells. Owing to its N-terminal nuclear localization signal, LKB1 is predominantly located in nucleus especially when it is not associated with other proteins. Upon activation, LKB1 translocates to cytoplasm where it complexes with STRAD (STE-related adapter) and MO25 (mouse protein 25). SIRT1 and SIRT3 have been shown to deacetylate LKB1 leading to an increase in its cytoplasmic localization, binding with STRAD and MO25 and activation of kinase function [40, 41]. We show that HNK increases the expression of SIRT1 and SIRT3 and overexpression of SIRT1 and SIRT3 increases the cytoplasmic localization of LKB1 in breast cancer cells. These results support a role for SIRT1/3 in HNK-mediated LKB1 activation. Although a role of LKB1 in cancer cell EMT has been proposed in cancer cells [49], the underlying molecular mechanisms remain elusive. We discovered that LKB1 plays an important role in HNK-mediated upregulation of miR-34a expression in breast cancer cells. This is an important finding as miR-34a is involved in tumor suppression by inhibiting various genes regulating cell proliferation, migration, invasion and EMT in many cancer types including breast cancer [50]. Our study shows that miR-34a is not only vital for HNK-mediated inhibition of EMT, and stemness but also plays an important role in HNK-mediated inhibition of leptin-function. Considering that miRs are promising candidates as biomarkers owing to their low complexity, stability, and ease of amplification and quantification, miR-34a can potentially serve as a biomarker for HNK function.

The realization of the impact of hyperleptinemia associated with obese state on breast cancer incidence, behavior, and prognosis, has spurred a great interest in the development of effective strategies to inhibit multipartite leptin signaling and oncogenic functions. Various approaches have been proposed to neutralize leptin activity either by directly targeting leptin or deactivating leptin receptor, for example, soluble leptin receptors (LRs), leptin peptidomimetics (LPA-2), synthetic leptin-antagonists, and anti-LR monoclonal antibodies (anti-LR mAbs) [17, 51–53]. Leptin receptor antagonists, Aca 1 (aa 121–129; modifications Nva123, Aca129) and Allo-aca (aa 121–129; modifications alloThr121, Nva123, Aca129) have been shown to inhibit leptin-dependent growth and signaling in various cancer cells [54, 55]. In addition, Stat3 inhibitors (chemical and bioactive) have been shown to block leptin signaling [56]. It is well-recognized that an ideal strategy to inhibit oncogenic effects of leptin should be safe, highly efficacious, and should lack toxicity to allow long-term use. Our study showed the potential of HNK as an effective and non-toxic inhibitor of oncogenic effects of leptin. HNK is particularly encouraging as it exhibits desirable spectrum of bioavailability in contrast with many other natural products [57]. While poor absorption and rapid excretion has marred the development of many other polyphenolic agents [58], HNK does not have these deficiencies. It has been shown that HNK can cross the blood-brain barrier and significant systemic levels of HNK can be achieved in preclinical models [59]. Owing to these qualities, HNK is a promising bioactive agent to be developed as leptin-antagonist.

In conclusion, we uncovered a novel role of HNK as an inhibitor of leptin-induced EMT and stemness, which involves LKB1 and miR-34a. Our results demonstrate the integral role of a previously unrecognized functional crosstalk between LKB1 and miR-34a in facilitating HNK-mediated inhibition of oncogenic actions of leptin.

## MATERIALS AND METHODS

### Ethics statement

The investigation was conducted in accordance with the ethical standards and guidelines approved by the authors’ institutional review board (Johns Hopkins University IACUC).

### Cell culture and reagents

The human breast cancer cell lines, MCF7, MDA-MB-468, SUM149, SUM159, T47D, SKBR3 and MDA-MB-231 were obtained from the American Type Culture Collection (ATCC, Manassas, VA), resuscitated from early passage liquid nitrogen vapor stocks as needed and cultured according to supplier's instructions. Cell line authentication was done by analysis of known genetic markers or response (e.g., expression of estrogen receptor and p53 and estrogen responsiveness). Cells were cultured for less than 3 months before reinitiating cultures and were routinely inspected microscopically for stable phenotype. For treatment, cells were seeded at a density of 1 × 10^6^ /100-mm tissue culture dish. After 16 hours of serum starvation, the culture media were changed to serum free media containing treatments as indicated. Cells were treated with 100 ng/ml human recombinant leptin (Sigma, St. Louis, MO). We extracted Honokiol (HNK) from seed cone of *Magnolia grandiflora* according to our previously published study [60]. Previous studies from our lab have shown that 25 μM honokiol inhibits cell viability, and cell proliferation while 5 μM honokiol doesn't affect cell viability and proliferation. Accordingly, we used 5 μM honokiol to examine the effect of honokiol on mammosphere formation, migration, and invasion [28, 29][30]. Antibodies for Slug (C19G7), Snail (L70G2), Zeb2 (ab25837), Nanog (D73G4), Oct4 (#2750), Sox2 (D6D9), LKB1 (D60C5), SIRT1 (C14H4), SIRT3 (C73E3), E-cadherin (24E10), and Occludin (D15G7) were purchased from Cell Signaling Technology (Danvers, MA). β-Actin antibody was purchased from Sigma-Aldrich (St. Louis, MO). Synthetic miRNA miR-34a mimic and siRNA were purchased from Applied Biosystems (Ambion, Austin, TX). pcDNA3-Flag-LKB1-wild-type (LKB1-WT), Flag-SIRT1, pcDNA3.1-Flag-SIRT3 plasmid constructs were procured from Addgene [61–63].

### Western blotting

Whole cell lysate was prepared by scraping breast cancer cells in 250 μl of ice cold modified RIPA buffer [64]. Equal amount of lysate protein was resolved on sodium-dodecyl sulfate polyacrylamide gel, transferred to nitrocellulose membrane, and western blot analysis was performed. Immunodetection was performed using enhanced chemiluminescence (ECL system, Amersham Pharmacia Biotech Inc., Arlington Heights, IL) according to manufacturer's instructions.

### RNA isolation, miR, transfection and RT-PCR

For RNA isolation and RT-PCR, total cellular RNA was extracted using the TRIzol Reagent (Life Technologies, Inc., Rockville, MD). RT-PCR was performed using specific sense and antisense PCR primers. Cells were transfected with miR-34a mimic or miR-34a inhibitor or control-miR (Applied Biosystems, Ambion, Austin, TX) using Fugene transfection reagent (Promega Corporation, Madison, WI). Standardization of miR-34a mimic and inhibitor is shown in supplementary figure 4. For qRT-PCR detection of miR-34a, miRNA-specific RT-primers (assay IDs: hsa-miR-34a, 000426), TaqMan miRNA Assay (Applied Biosystems, Ambion, Austin, TX) and Platinum Taq Polymerase Reagents (Invitrogen, Grand Island, NY) were used. Data were calculated by using the standard ΔΔ*Ct* method and microRNA expression was represented as fold-difference of each treatment *vs*. vehicle-treated control. Statistics was performed by using one-way ANOVA and Student's *t*-test post-hoc analysis. Statistical significance was accepted when p was <0.05. pcDNA3-Flag-LKB1-wild-type (LKB1-WT), Flag-SIRT1, pcDNA3.1-Flag-SIRT3 plasmid constructs were transfected using Fugene 6 (Promega Corporation, Madison, WI) transfection reagent.

### Immunofluorescence and confocal imaging

Breast cancer cells were subjected to immunofluorescence analysis. Fixed and immunofluorescently stained cells were imaged using a Zeiss LSM510 Meta (Zeiss) laser scanning confocal system configured to a Zeiss Axioplan 2 upright microscope. All experiments were performed multiple times using independent biological replicates.

### Scratch-migration assay

To perform migration assays [12, 64]; cells were plated into the 6-well cell culture plate. Cells were allowed to grow in DMEM containing 10% FBS to confluence, and then were washed with serum-free medium and serum starved for 16 h. A 1-mm wide scratch was made across the cell layer using a sterile pipette tip. Plates were photographed immediately after scratching. Cells were treated with human recombinant leptin at 100 ng/ml and/or HNK at 5 μM alone and in combination. Plates were photographed after 8 h, 24 h and 48 h at the identical location of the initial image.

### Mammosphere assays

were performed as previously described [34] and spheres (>50 μm) were counted [65].

### Preparation of subcellular fractions

Cellular cytosolic and nuclear fractions were prepared by incubating cells in 100 μl of ice-cold lysis buffer [10 mM Tris-Hcl (pH 7.4), 10 mM NaCl, 3 mM MgCl_2_, 0.5% NP-40, 2 mM DTT and 0.1 mM PMSF]. The lysates were incubated for 5min on ice followed by centrifugation at 4,000g for 10 min at 4°C to precipitate nuclei. Supernatant was stored as cytoplasmic fraction. Nuclear pellet was incubated with 100 μl of ice-cold extraction buffer [20 mM Tris-Hcl (pH 7.9), 0.42M KCl, 0.2 mM EDTA, 10% Glycerol, 2 mM DTT and 0.1 mM PMSF] for 10min followed by centrifugation at 12,000g for 10min at 4°C to clear the nuclear debris. Total protein was quantified using the Bradford protein assay kit (Biorad, Hercules, CA). Equal amount of protein was subjected to western blot analysis.

### LKB1 stable knockdown using lentiviral short hairpin RNA

Five pre-made lentiviral LKB1 short hairpin RNA (shRNA) constructs and a negative control construct created in the same vector system (pLKO.1) were purchased from Open Biosystems (Huntville, AL). Constructs were used for transient transfection using Fugene or Lipofectamine. Paired LKB1 stable knockdown cells (MCF7) were generated following our previously published protocol [66].

### Breast Tumorigenesis assay

MDA-MB-231 cells xenografts were generated; as previously described [28], grouped in four experimental groups. Mice were treated with intraperitoneal (IP) injections of 1) control (saline and Intralipid); 2) HNK, at 3 mg/mouse/day in 20% Intralipid (Baxter Healthcare, Deerfield, IL), three times per week; 3) recombinant leptin (dosage of 5 mg/kg), 5 days a week; 4) leptin and HNK for 4 weeks. The dose and route of HNK and leptin administration was selected from our previous studies documenting *in vivo* efficacy of honokiol and leptin [17, 28]. Tumors were regularly measured; collected after 4 weeks of treatment, weighed, and subjected to further analysis. These tumors were utilized for examining the expression of Vimentin, Fibronectin, Zeb1, Zeb2, Slug and actin. All animal studies were in accordance with the guidelines of Johns Hopkins University IACUC.

### Statistical analysis

All experiments were performed thrice in triplicates. Statistical analysis was performed using Microsoft Excel software. Significant differences were analyzed using student's *t* test and two-tailed distribution. Results were considered to be statistically significant if *p* < 0.05. Results were expressed as mean ± SE between triplicate experiments performed thrice.

## SUPPLEMENTARY MATERIAL FIGURES


