# Supplementary material for: Honokiol activates LKB1-miR-34a axis and antagonizes the oncogenic actions of leptin in breast cancer
Source: Oncotarget. 2015 Aug 24;6(30):29947–62. doi: 10.18632/oncotarget.4937 (PMC4745774; doi:10.18632/oncotarget.4937)
Supplement: Supplementary file 1 [file oncotarget-06-29947-s001.pdf]

## SUPPLEMENTARY FIGURES

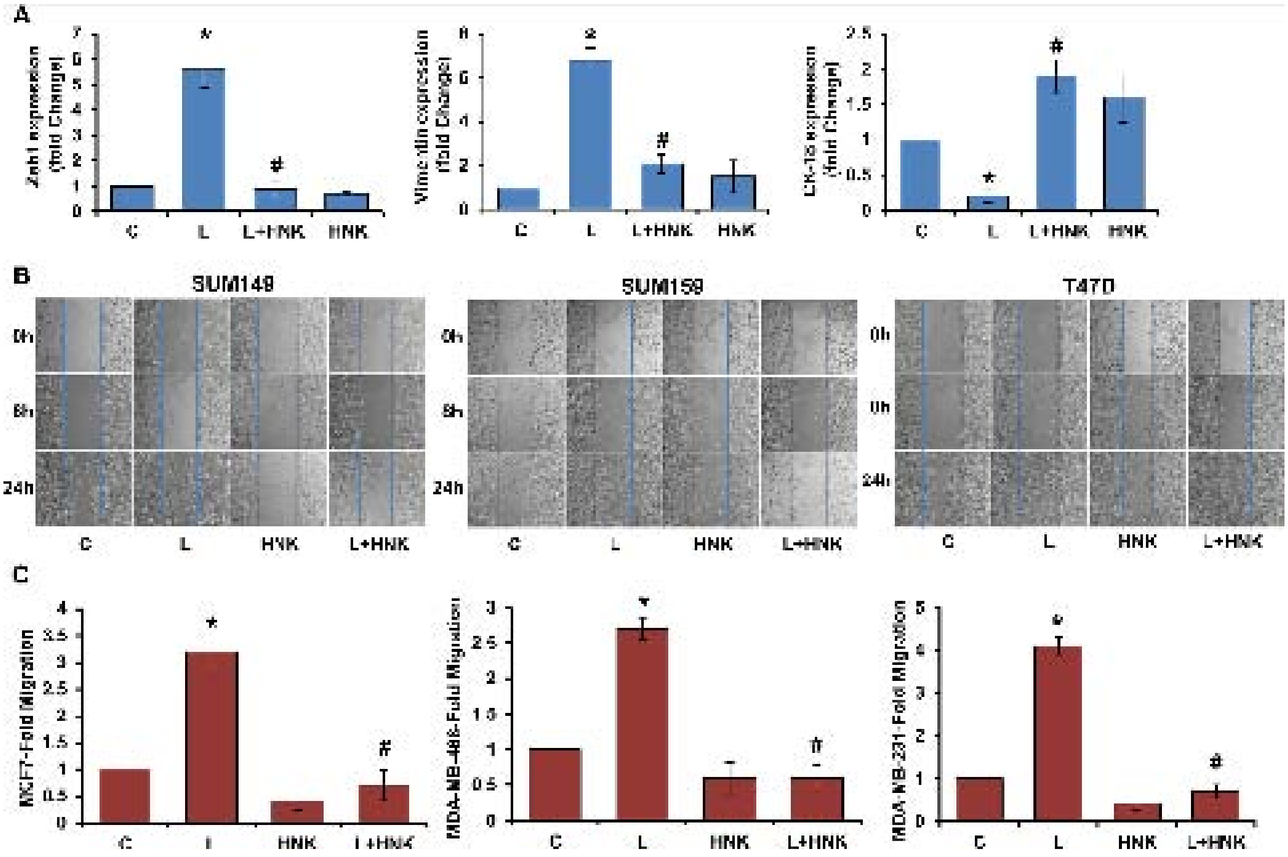


**Supplementary Figure S1: Honokiol modulates the expression of epithelial and mesenchymal marker genes and inhibits leptin-induced migration of breast cancer cells. A.** MCF7 cells were treated with leptin (L) (100 ng/ml) and/or Honokiol (HNK) (5 µM) as indicated, total RNA was isolated and expression levels of Zeb1, vimentin and CK-18 were analyzed. Actin was used as control. Bar graphs represent the fold-change in the expression of Zeb1, vimentin and CK-18. *$p < 0.001$, compared with untreated controls. #$p < 0.001$, compared with leptin-alone treatment. **B.** SUM149, SUM159 and T47D cells were treated with leptin (L, 100 ng/ml) and/or honokiol (HNK, 5 µM) alone and in combination as indicated and subjected to scratch-migration assay. **C.** MCF7, MDA-MB-468 and MDA-MB-231 cells were treated with leptin (L, 100 ng/ml) and/or honokiol (HNK, 5 µM) alone and in combination as indicated and subjected to scratch-migration assay. Bar graphs show the fold-change in migration of breast cancer cells. *$p < 0.001$, compared with untreated controls. #$p < 0.01$, compared with leptin-alone treatment.

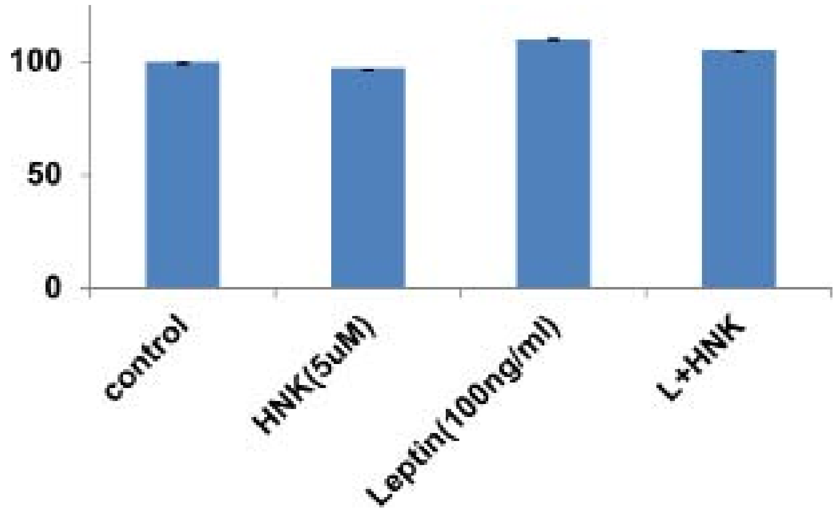


**Supplementary Figure S2: Honokiol does not alter growth of MCF10A cells.** MCF10A cells were treated with leptin (L) (100 ng/ml) and/or honokiol (HNK) (5 μM) as indicated and subjected to SRB growth assay.

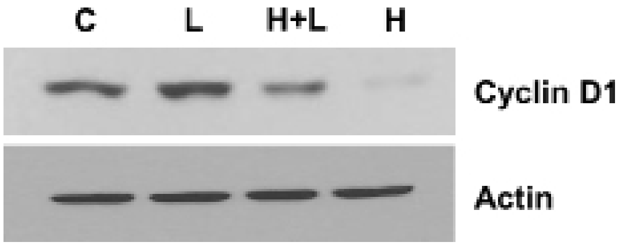


**Supplementary Figure S3: Honokiol decreases the expression of leptin-induced cyclin D1.** MCF7 cells were treated with leptin (L) (100 ng/ml) and/or honokiol (HNK) (5 μM) as indicated, total protein was isolated followed by immunoblot analysis for cyclin D1 expression.

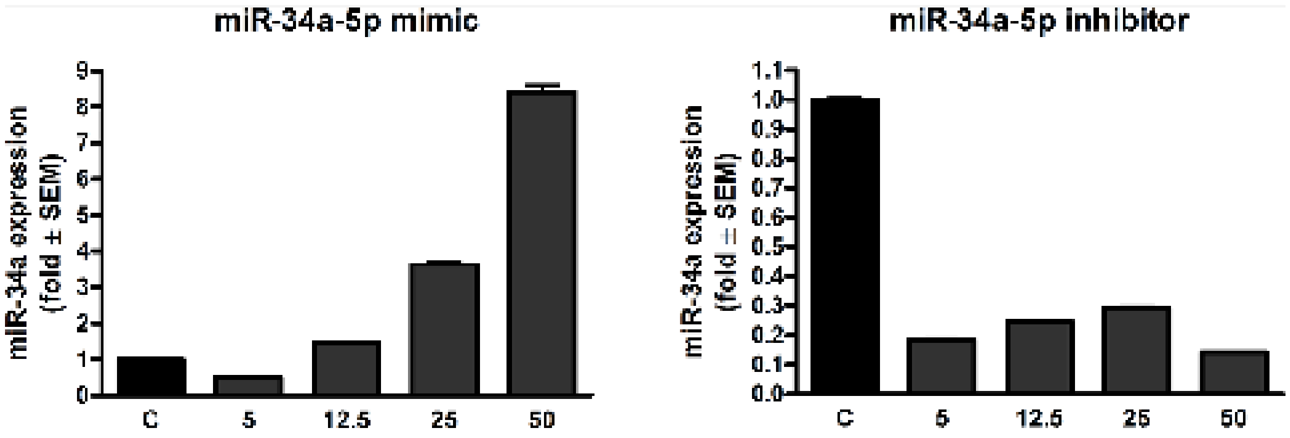


**Supplementary Figure S4: miR-34a mimic and miR-34a inhibitor.** MCF7 cells were transfected with miR-34a inhibitor or miR-34a mimic as indicated. The miR-34a levels were measured.
